# Supplementary figures and images for: Enhanced Bioaccessibility and Antioxidant Activity of Curcumin from Transglutaminase Cross-Linked Mulberry Leaf Protein-Stabilized High-Internal-Phase Pickering Emulsion: In Vivo and In Vitro Studies
Source: Foods. 2024 Dec 6;13(23):3939. doi: 10.3390/foods13233939 (PMC11641251; doi:10.3390/foods13233939)

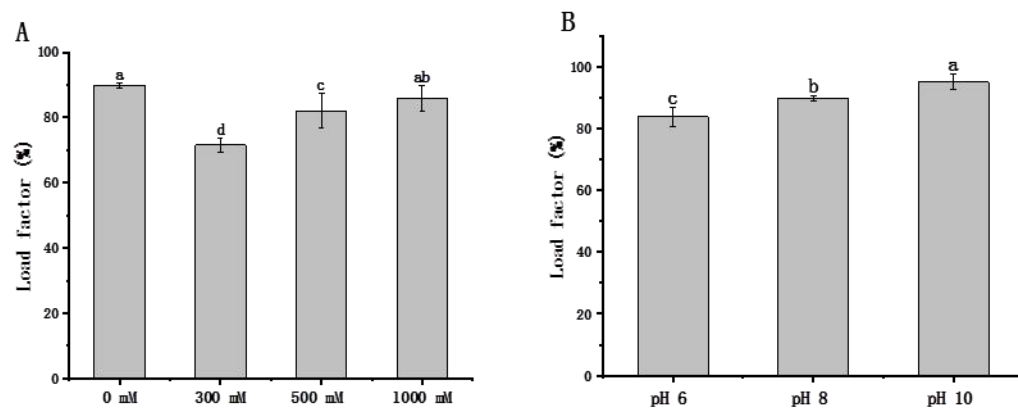

**Fig.S1** Load factor of curcumin by HPIEs prepared at different ion concentrations (A) and pH (B).

Supplement: Supplementary file 1 [file foods-13-03939-s001.zip › foods-3313036-supplementary.pdf]
